# Excess enthalpy of mixing of mineral solid solutions derived from density-functional calculations

**DOI:** 10.1007/s00269-020-01085-8

**Published:** 2020-02-17

**Authors:** Artur Benisek, Edgar Dachs

**Affiliations:** Chemistry and Physics of Materials, University of Salzburg, Jakob-Haringer-Str. 2a, 5020 Salzburg, Austria

**Keywords:** Thermodynamics, Heat of mixing, First principles, Ab initio, DFT, CASTEP, MgO–CaO, Halides, Garnets, Feldspars, Pyroxenes, Amphiboles

## Abstract

Calculations using the density-functional theory (DFT) in combination with the single defect method were carried out to determine the heat of mixing behaviour of mineral solid solution phases. The accuracy of this method was tested on the halite–sylvite (NaCl–KCl) binary, pyrope–grossular garnets (Mg_3_Al_2_Si_3_O_12_–Ca_3_Al_2_Si_3_O_12_), MgO–CaO (halite structure) binary, and on Al/Si ordered alkali feldspars (NaAlSi_3_O_8_–KAlSi_3_O_8_); as members for coupled substitutions, the diopside–jadeite pyroxenes (CaMgSi_2_O_6_–NaAlSi_2_O_6_) and diopside–CaTs pyroxenes (CaMgSi_2_O_6_–CaAlAlSiO_6_) were chosen for testing and, as an application, the heat of mixing of the tremolite–glaucophane amphiboles (Ca_2_Mg_5_Si_8_O_22_(OH)_2_–Na_2_Mg_3_Al_2_Si_8_O_22_(OH)_2_) was computed. Six of these binaries were selected because of their experimentally well-known thermodynamic mixing behaviours. The comparison of the calculated heat of mixing data with calorimetric data showed good agreement for halite–sylvite, pyrope–grossular, and diopside–jadeite binaries and small differences for the Al/Si ordered alkali feldspar solid solution. In the case of the diopside–CaTs binary, the situation is more complex because CaTs is an endmember with disordered cation distributions. Good agreement with the experimental data could be, however, achieved assuming a reasonable disordered state. The calculated data for the Al/Si ordered alkali feldspars were applied to phase equilibrium calculations, i.e. calculating the Al/Si ordered alkali feldspar solvus. This solvus was then compared to the experimentally determined solvus finding good agreement. The solvus of the MgO–CaO binary was also constructed from DFT-based data and compared to the experimentally determined solvus, and the two were also in good agreement. Another application was the determination of the solvus in tremolite–glaucophane amphiboles (Ca_2_Mg_5_Si_8_O_22_(OH)_2_–Na_2_Mg_3_Al_2_Si_8_O_22_(OH)_2_). It was compared to solvi based on coexisting amphiboles found in eclogites and phase equilibrium experiments.

**Electronic supplementary material:**

The online version of this article (10.1007/s00269-020-01085-8) contains supplementary material, which is available to authorized users.

## Introduction

This contribution is a continuation of our former study (Benisek and Dachs 2018), where we calculated internal energies and entropies of 21 well-known endmembers using the density-functional theory (DFT) and transformed them into standard enthalpies of formation from the elements and standard entropies enabling a direct comparison with the measured quantities. These data were then integrated into existing thermodynamic data sets, making them available to a broad range of applications (Benisek and Dachs 2018). Since many mineral phases are solid solutions, thermodynamic mixing properties are essential for any petrologically relevant phase equilibrium calculations.

The enthalpy of a solid solution at a particular composition (*H*_(*X*)_) may deviate from the behaviour of a mechanical mixture, i.e. from the linear combination of the enthalpies of the endmembers A and B (*H*^mechmix^):

1 $$ H^{{{\text{mechmix}}}} = \, \left( {X_{{\text{A}}} H^{{\text{A}}} + X_{{\text{B}}} H^{{\text{B}}} } \right). $$

*H*^A^, *H*^B^, *X*_A_, and *X*_B_ are the enthalpies and the mole fractions of the A and B components, respectively. This deviation is called the excess enthalpy of mixing or the heat of mixing (∆*H*^mix^):

2 $$ \Delta H^{{{\text{mix}}}} = H_{(X)} {-} \, \left( {\left( {{1} - X_{{\text{B}}} } \right)H^{{\text{A}}} + X_{{\text{B}}} H^{{\text{B}}} } \right). $$

∆*H*^mix^ is a substantial thermodynamic property describing the behaviour of solid solutions. This property is responsible for ordering and exsolution phenomena, e.g. exsolution lamellae in feldspars (perthite), in calcite–dolomite and in pyroxene solid solutions; ordering in omphacite. ∆*H*^mix^ data can be measured experimentally by solution calorimetry (e.g. Navrotsky 1997; Hovis 2017; Benisek et al. 2003; Carpenter et al. 1985; Newton et al. 1977), which is a rather time-consuming technique. These data are then described by a mixing model using so-called interaction parameters (*W*_AB_ and *W*_BA_), e.g. the Margules mixing model:

3 $$ \Delta H^{{{\text{mix}}}} = \, \left( {{1} - X_{{\text{B}}} } \right)X_{{\text{B}}}^{2} W_{{{\text{AB}}}} + \, \left( {{1} - X_{{\text{B}}} } \right)^{{2}} X_{{\text{B}}} W_{{{\text{BA}}}} . $$

Such a description is necessary to define the activity–composition relations, which are then needed for petrological investigations (e.g. geothermometry, calculations of equilibrium phase diagrams).

However, ∆*H*^mix^ can also be calculated by ab initio methods (e.g. Ozolins et al. 1997). A particularly effective way of calculating ∆*H*^mix^ of a disordered phase at any intermediate composition is the single defect method of Sluiter and Kawazoe (2002). This method makes use of a single substitutional defect incorporated into a supercell of a host endmember. The energy calculations for the endmembers and for such supercells provide direct knowledge of the interaction parameters, because the results can be easily transformed into the slopes of the heat of mixing functions in the limits of *X*_B_ = 0 and *X*_B_ = 1 (Li et al. 2014). The ∆*H*^mix^ calculated by the single defect method represents that of a disordered solid solution as discussed by many studies (e.g. Vinograd and Sluiter 2006; Vinograd and Winkler 2010; Vinograd et al. 2013; Li et al. 2014; Vinograd et al. 2018).

In this study, the single defect method was tested on the following solid solutions: halite–sylvite (NaCl–KCl), pyrope–grossular (Mg_3_Al_2_Si_3_O_12_–Ca_3_Al_2_Si_3_O_12_), MgO–CaO (with halite structure), Al/Si ordered alkali feldspar (NaAlSi_3_O_8_–KAlSi_3_O_8_), and two binaries characterised by coupled substitutions, i.e. diopside–jadeite pyroxenes (CaMgSi_2_O_6_–NaAlSi_2_O_6_) and diopside–CaTs pyroxene (CaMgSi_2_O_6_–CaAlAlSiO_6_).

These six binaries were chosen because their thermodynamic mixing behaviours are well known from calorimetric or phase equilibrium experiments, enabling a direct comparison of calculated with experimentally derived mixing behaviours and thus an assessment if DFT in combination with the single defect method can provide reasonable results. The enthalpic mixing behaviour of the halite–sylvite binary was investigated by solution calorimetry at 298 K in the work of Barrett and Wallace (1954) and that of the pyrope–grossular binary by high-temperature solution calorimetry (Newton et al. 1977). ∆*H*^mix^ of the alkali feldspar binary studied by Hovis (1988) was reinvestigated in 2017 by HF solution calorimetry by the same author on a large number of specimens producing well-defined heat of Na–K mixing data (Hovis 2017). ∆*H*^mix^ of the diopside–jadeite binary was measured by high-temperature solution calorimetry (Wood et al. 1980). The same method was used to measure ∆*H*^mix^ of the diopside–CaTs solid solution by Newton et al. (1977) and reinvestigated by Benisek et al. in 2007, who found results consistent with those of the former study.

The calculated ∆*H*^mix^ of the Al/Si ordered alkali feldspar binary in combination with measured vibrational entropy of mixing data (∆*S*_vib_^exc^) was then applied to calculate the corresponding solvus, allowing a comparison with the one studied experimentally by the ion exchange and homogenisation–unmixing techniques (Bachinski and Müller 1971). A more sophisticated procedure that calculated both ∆*H*^mix^ and ∆*S*_vib_^exc^ using DFT methods was applied to the MgO–CaO binary. The solvus in this join was determined independently by many researchers (e.g. Doman et al. 1963) via phase equilibrium experiments, all of which were modelled by Yin and Argent (1993). As an application of determining ∆*H*^mix^ of a binary for which no calorimetric data exist, tremolite–glaucophane amphiboles were chosen. The resulting data, together with estimated entropy data, were used to construct the solvus and to compare it with solvi obtained from naturally coexisting amphiboles found in different eclogites (Reynard and Ballevre 1988) and from phase equilibrium experiments (Jenkins et al. 2014).

## Experimental methods

### Computational methods

Quantum mechanical calculations were based on the DFT plane wave pseudopotential approach implemented in the CASTEP code (Clark et al. 2005) included in the Materials Studio software from Biovia^®^. The calculations used the local density approximation (LDA) for the exchange–correlation functional (Ceperley and Alder 1980) and norm-conserving pseudopotentials to describe the core–valence interactions. For the k-point sampling of the investigated unit cells, a Monkhorst–Pack grid (spacing of 0.03/Å) was used (Monkhorst and Pack 1976) and convergence was tested by performing calculations using a denser k-point grid. The structural relaxation was calculated by applying the BFGS algorithm (Pfrommer et al. 1997), where the convergence threshold for the force on an atom was 0.01 eV/Å. In addition to the LDA calculations, the gradient corrected functional (GGA-PBE, Perdew et al. 1996) and its revised form for solids (GGA-PBESOL, Perdew et al. 2008) were used in some cases.

### Single defect method to calculate the heat of mixing (∆*H*^mix^)

In a first step, the internal energies (*E*) of the unit cells of the endmembers were calculated and normalised to one formula unit. Next, large supercells were generated containing one single substitutional defect. In the case of the pyrope–grossular solid solution, for example, the largest supercell contained 47 Ca plus 1 Mg on the dodecahedral positions, which is equal to the size of 16 formula units and a mole fraction of *X*_Gr_ = 47/48. Other supercells of this binary contained 23 Mg plus 1 Ca and 23 Ca plus 1 Mg on the dodecahedral positions, resulting in mole fractions of *X*_Gr_ = 1/24 and 23/24, respectively. The internal energies of these supercells were then calculated and normalised to one formula unit. The excess internal energy of mixing (∆*E*^mix^), i.e. the deviation from the linear combination of the internal energies of the endmembers, was calculated according to Eq. 2.

∆*E*^mix^ and ∆*H*^mix^ are related by the volume term *P* ∆*V*^mix^. ∆*V*^mix^ is typically less than 1 J/bar/mol (e.g. Geiger 2001), whereas ∆*E*^mix^ is in the range of several kJ/mol. At a pressure of 1 bar, where the calculated ∆*E*^mix^ is compared to the measured ∆*H*^mix^, the volume term *P* ∆*V*^mix^ can be neglected, i.e.

4 $$ \Delta E^{{{\text{mix}}}} \approx \Delta H^{{{\text{mix}}}} . $$

### DFT calculations of the vibrational excess entropy of mixing (∆*S*_vib_^exc^)

The vibrational excess entropy of mixing (∆*S*_vib_^exc^) is defined as the deviation from the linear combination of the vibrational entropies of the endmembers (similar to ∆*H*^mix^, see Eq. 2). To calculate ∆*S*_vib_^exc^ of a solid solution by DFT methods, investigations of large enough cells are necessary, as shown on Cu_3_Au by Benisek et al. (2018). Otherwise, the results depend too strongly on the used atomic configuration. This situation occurred with Cu_3_Au when investigating cells with only eight crystallographic sites on which substitutions took place. To simulate the random character of a solid solution, cells with 32 sites on which substitutions took place were necessary (Benisek et al. 2018).

### Calculation of the solvus from DFT data

To model the solvus from thermodynamic data, the heat of mixing, ∆*H*^mix^, the excess vibrational entropy of mixing, ∆*S*_vib_^exc^, and the configurational entropy, *S*^config^, have to be known (e.g. Benisek and Dachs 2013; Benisek et al. 2014). Whereas ∆*H*^mix^ and ∆*S*_vib_^exc^ can be calculated directly using DFT methods, the calculation of the excess configurational entropy (i.e. the deviation from the fully disordered state due to short-range ordering—SRO) would need additional methods, e.g. the cluster expansion method (Vinograd et al. 2009; Vinograd and Winkler 2010), which was not applied in this work. An estimate of the configurational excess entropy is nevertheless possible by comparing experimentally derived *T*–*X* positions of solvi (with samples that are characterised by SRO) with those calculated using DFT derived thermodynamic data (valid for the fully disordered state).

The *T*–*X* position of a solvus is calculated by finding those two compositions, for a given temperature and pressure, at which the chemical potentials of components A and B in the two coexisting phases, ph1 and ph2, forming the miscibility gap, are equal:

5 $$ \mu_{{\text{A}}}^{{{\text{ph1}}}} = \mu_{{\text{A}}}^{{{\text{ph2}}}} \;{\text{and}} $$

6 $$ \mu_{{\text{B}}}^{{{\text{ph1}}}} = \mu_{{\text{B}}}^{{{\text{ph2}}}} . $$

## Results and discussion

### Halite–sylvite binary

**Fig. 1:**
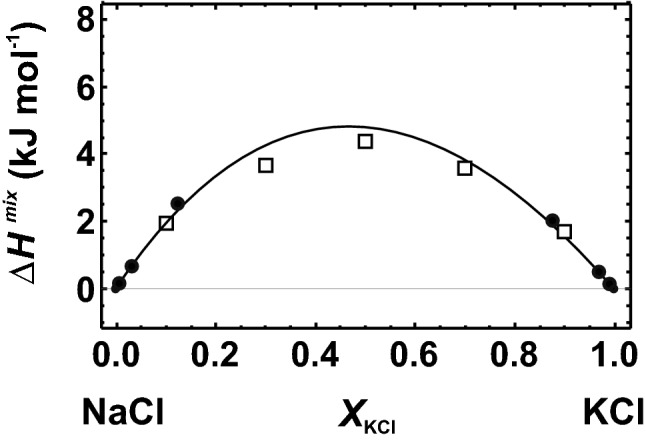
Heat of mixing (∆*H*^mix^) of the halite (NaCl)–sylvite (KCl) binary. Experimental data from Barrett and Wallace (1954) are indicated by open squares. Their experimental errors (1 sd) are smaller than plot symbols. Closed circles represent the results from LDA calculations. The results using large supercells at *X*_KCl _≈ 0.03 and 0.97 were fitted by a Margules mixing model (solid line). The LDA results from smaller and larger supercells are also shown

In the halite–sylvite (NaCl–KCl) solid solution, Na and K are octahedrally coordinated by Cl atoms. To model the solid solutions, two large supercells containing 31 Na and 1 K on the Na-rich side and 31 K and 1 Na on the K-rich side were constructed. The cells had 32 formula units (FU) and mole fractions of *X*_KCl_ = 1/32 and 31/32, respectively. The resulting ∆*H*^mix^ data were fitted to a Margules mixing model enabling a comparison with the measured data. As illustrated in Fig. 1, the calculated ∆*H*^mix^ values agree well with the experimental values. Very small deviations between calculated and measured ∆*H*^mix^ values can be found in the intermediate part of the solid solution. Here, experimental difficulties occur in producing samples without exsolutions (Barrett and Wallace 1954). Principally, the calculated ∆*H*^mix^ values depend on the size of the supercells. We, therefore, investigated additionally smaller supercells with only 8 FU and larger supercells with 108 FU. These data are presented in the supplementary materials (Supplementary Appendix A) and show that the single defects should be separated by a distance of at least ca. 12 Å (single defects of neighbouring cells), which is the case for supercells containing 32 FU. The supercells with only 8 FU are characterised by distances between the single defects of ca. 8 Å. They produced significantly higher ∆*H*^mix^ values compared to the larger supercells.

The NaCl–KCl binary is characterised by positive vibrational excess entropies as measured by calorimetry by Benisek and Dachs (2013). Using these data, the Gibbs free energy of mixing and in consequence the halite–sylvite solvus were calculated showing good agreement with literature data (Barrett and Wallace 1954; Vesnin and Zakovryashin 1979). A comparison is given in the supplementary materials (Supplementary Appendix B).

### Pyrope–grossular solid solution

**Fig. 2:**
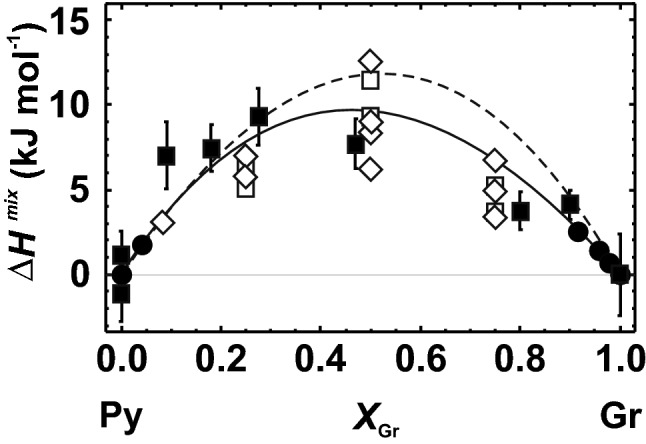
Heat of mixing (∆*H*^mix^) of the pyrope (Py)–grossular (Gr) solid solution. Experimental data from Newton et al. (1977) are indicated by solid squares with error bars (1 sd). Closed circles represent ∆*H*^mix^ form LDA calculations using the single defect method and are modelled applying a Margules mixing model (solid line). Broken line shows the Margules fit to the results of GGA-PBE calculations (single defect). Open squares and diamonds: ∆*H*^mix^ derived from GGA-PBESOL and LDA calculations that are not based on the single defect method, but use different configurations in cells with intermediate composition

This binary is characterised by Mg–Ca mixing on one crystallographic position, the dodecahedral site. The single defect method using the LDA functional yielded ∆*H*^mix^ values in good agreement with the calorimetric data of Newton et al. (1977) as presented in Fig. 2. In addition to the LDA calculations, the gradient-corrected functional (GGA-PBE, Perdew et al. 1996) and its revised form for solids (GGA-PBESOL, Perdew et al. 2008) were used to calculate ∆*H*^mix^ of this solid solution. The GGA-PBE functional provided results that are larger than the calorimetric ∆*H*^mix^ values, especially in the Ca-rich region (broken line in Fig. 2). In Fig. 2, additional results (using LDA and GGA-PBESOL, but not the single defect method) are shown, obtained from cells with intermediate compositions, each having different Ca/Mg configurations demonstrating that the resulting ∆*H*^mix^ values of such cells depend strongly on the configuration used. The LDA and GGA-PBESOL functionals resulted in similar ∆*H*^mix^ values.

The ∆*H*^mix^ function using the LDA calculations (solid line in Fig. 2) is comparable to the results obtained by a cluster expansion method of Sluiter et al. (2004). The theoretical study of Vinograd and Sluiter (2006) proposed a temperature-dependent ∆*H*^mix^ behaviour for the pyrope–grossular solid solution. The ∆*H*^mix^ values at low temperatures (< 700 K) are significantly smaller than our LDA single defect results and those of Newton et al. (1977), but agree well at higher temperatures (~ 1500 K). At such high temperatures, the configurational entropy is that of a disordered solid solution (Vinograd and Sluiter 2006). The agreement of the ∆*H*^mix^ behaviour resulting from the single defect method with its high-temperature behaviour, as determined by Vinograd and Sluiter (2006), indicates that the single defect method yields ∆*H*^mix^ of a disordered solid solution.

### MgO–CaO solid solution

The MgO–CaO binary has a halite structure, where Mg and Ca are octahedrally coordinated by oxygen atoms. To the best of our knowledge, no heat of mixing data exist for this solid solution. However, many experimentally determined solvus data have been published for this binary, which were compiled and recalculated by Yin and Argent (1993). We therefore performed DFT calculations for both ∆*H*^mix^ and ∆*S*_vib_^exc^ to model the MgO–CaO solvus.

The results of the calculations with regard to enthalpy were described by a Margules mixing model using the interaction parameters *W*^*H*^_MgCa_ = 65.24 and *W*^*H*^_CaMg_ = 86.83 kJ/mol. For the calculations of ∆*S*_vib_^exc^, three cells with 64 atoms (24 Mg, 8 Ca, and 32 O) were investigated, differing in their atomic arrangements (disordered, clustered, or short-range ordered). All cells yielded almost identical results in accordance with the findings on Cu_3_Au (Benisek et al. 2018). Applying calorimetry and DFT calculations, these authors showed that the vibrational entropy did not depend on the degree of short-range ordering in Cu_3_Au. The latter method, however, requires the investigation of large enough cells containing at least 32 sites on which the substitution takes place. The independence of the vibrational entropy from the degree of short-range ordering may be explained by the following considerations: The geometry of distinct crystallographic sites, on which the substitution occurs, varies slightly from one site to the other in a disordered phase depending on the environment of the particular site. In a short-range ordered phase, there are sites where the environment of atom A is enriched with atom B (with different degrees of enrichment from one site to the other). The enthalpy and the configurational entropy of such short-range ordered structure differs from that of a fully disordered one. However, the phonons only see averaged crystallographic sites and hence the vibrational entropy tends to be independent of the degree of short-range ordering/clustering. This situation can be modelled in DFT calculations, if large enough cells are used in computing the dynamical matrix (Benisek et al. 2018). The resulting ∆*S*_vib_^exc^ values for the MgO–CaO binary were described using a Margules model yielding *W*^*S*^_vib_ = 10.8 J/mol/K.

**Fig. 3:**
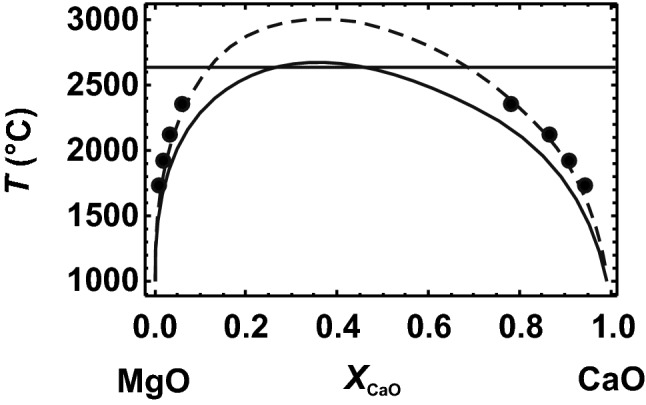
DFT-based solvus (solid line) of the MgO–CaO system (halite structure) at 1 bar compared to the experimentally determined solvus (solid circles) of Yin and Argent (1993). The broken line represents a calculated solvus using the same DFT data (∆*H*^mix^ and ∆*S*_vib_^exc^) plus a small reduction of the configurational entropy due to possible short-range clustering. At 2630 °C, this system is characterised by the eutectic interactions and are not shown in detail

Using the obtained mixing parameters, a solvus can be calculated and compared with the experimentally determined solvus. The calculated solvus is at slightly too low temperatures (Fig. 3). As found for the NaCl–KCl binary (Benisek and Dachs 2013) and for the alkali feldspar solid solutions (Benisek et al. 2014), samples equilibrated at the *P–T–X* conditions of the solvi are characterised by SRO. This situation, however, may be better described by short-range clustering; i.e. the environment of a Na atom is enriched in Na atoms, and that of a K atom is enriched in K atoms. Such an atomic distribution should be expected near the point where exsolution textures are developed. This short-range clustering reduces the configurational entropy from that of a fully disordered solid solution crystal. In the case of the NaCl–KCl binary, the decrease in the configurational entropy due to short-range clustering was described by an asymmetric Margules mixing model using *W*_NaK_^*S*^_config_ = 0 and *W*_KNa_^*S*^_config_ = − 3 J/mol/K (Benisek and Dachs 2013). In the case of the Al/Si disordered alkali feldspars, the decrease in the configurational entropy yielded *W*_NaK_^*S*^_config_ = 0 and *W*_KNa_^*S*^_config_ = − 7 J/mol/K (Benisek et al. 2014).

The experimentally determined MgO–CaO solvus can be perfectly reproduced, if the configurational entropy of a disordered atomic distribution is reduced by an amount given by a Margules mixing model with *W*_MgCa_^*S*^_config_ = − 2.5 and *W*_CaMg_^*S*^_config_ = − 3 J/mol/K (Fig. 3). These are quite realistic values and comparable to the situation in the NaCl–KCl and in the Al/Si disordered alkali feldspar solid solutions.

The vibrational entropy of the MgO–CaO solid solution was also calculated using a cell with only eight atoms (3 Mg, 1 Ca, and 4 O). The ∆*S*_vib_^exc^ value of this particular cell was more than twice as much as that of the cell with 64 atoms, emphasising the need for large cells in simulating the vibrational entropy of solid solutions.

### Al/Si ordered alkali feldspar solid solution

**Fig. 4:**
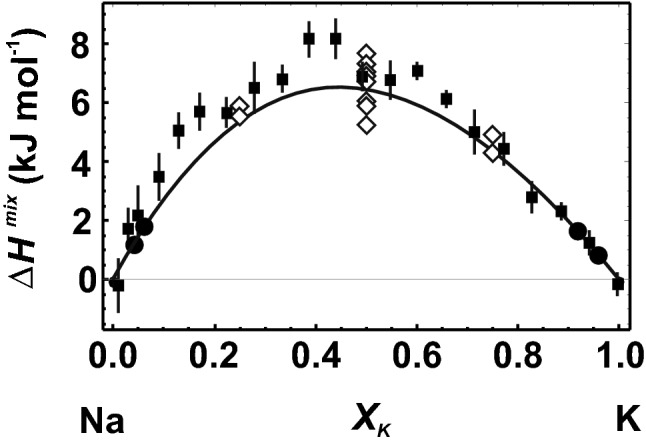
Heat of mixing (∆*H*^mix^) of the low albite (Na)–microcline (K) binary. The experimental data of Hovis (2017) are shown by solid squares with error bars (1 sd). Closed circles and solid line show the results from DFT calculations using the single defect method. Open diamonds represent results from DFT calculations using cells with different Na–K configurations

In alkali feldspars, Na–K mixing takes place on one crystallographic site, the irregular cavities in the tetrahedral framework. The DFT-based ∆*H*^mix^ data for Na–K mixing in Al/Si ordered feldspars are shown in Fig. 4, where they are compared to results from thoroughly investigated samples using HF calorimetry (Hovis 2017). The single defect DFT results are slightly lower than those of the calorimetric data, especially in the Na-rich region. In Fig. 4, additional results are shown (data in the intermediate compositional region, computed without using the single defect method). As was the case with the data for the pyrope–grossular binary, such an approach yields ∆*H*^mix^ values that depend strongly on the chosen configuration causing a scatter of 2–3 kJ/mol in ∆*H*^mix^.

**Fig. 5:**
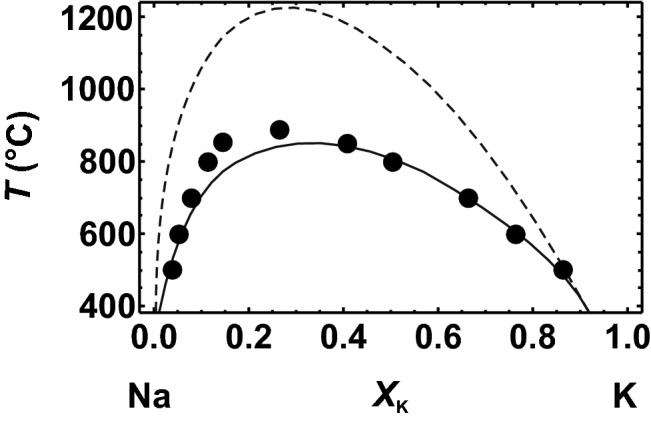
Solvus of the Al/Si ordered alkali feldspar binary, low albite (Na)–microcline (K), at 1 bar. Solid circles show mean solvus data experimentally determined by Bachinski and Müller (1971). Solid line shows the miscibility gap calculated using ∆*H*^mix^ derived from the single defect method of this study. Broken line represents the solvus using ∆*H*^mix^ from Hovis (2017). In both cases, the behaviour of the vibrational entropy of mixing was calculated using an interaction parameter of *W*^*S*^ = 9.1 J/mol/K (Benisek et al. 2014), which agrees with that of Haselton et al. (1983). The volume–composition behaviour does not play any role at 1 bar and is not considered

Using a Margules mixing model for the vibrational entropy with *W*^*S*^_vib_ = 9.1 J/mol/K, which is based on calorimetrically measured ∆*S*_vib_^exc^ values (Benisek et al. 2014), the solvus for this binary was calculated by using either the calorimetric or the DFT-based ∆*H*^mix^ data. In Fig. 5, both solvi are shown allowing comparison to the experimentally determined solvus of Bachinski and Müller (1971). The solvus calculated with DFT-based ∆*H*^mix^ has a slightly lower critical temperature than the experimentally determined solvus. Similar behaviour was found in the NaCl–KCl (Benisek and Dachs 2013), the Al/Si disordered alkali feldspar (Benisek et al. 2014), and the MgO–CaO binaries (this study). The difference for the Al/Si ordered alkali feldspar system is, though, small compared to those for the other binaries. Nevertheless, all binaries studied in this way (i.e. comparing solvi that are based on ∆*H*^mix^ and ∆*S*_vib_^exc^ values with experimentally determined ones) show similar characteristics, specifically, the need for excess configurational entropies to obtain agreement. The excess configurational entropies can be logically traced back to short-range clustering present in samples lying on the solvus. Such short-range clustering can be modelled using the configurational entropy of a fully disordered state plus a Margules mixing model using negative interaction parameters to describe the excess configurational entropy.

The calorimetrically based solvus of the Al/Si ordered alkali feldspars is, on the other hand, positioned at considerably higher temperatures than the experimentally determined solvus (Fig. 5). Positive excess configurational entropies would be required to achieve agreement, which is, however, not easy to explain. Hovis (2017) provided some explanations for such excess configurational entropies possibly present in his samples. At this point, note also that the solvus temperatures respond very sensitively to small changes in ∆*H*^mix^. The differences between calorimetric and DFT-based ∆*H*^mix^ data are only ~ 1 kJ/mol, but produce solvi that differ by almost 400 °C. Small structural changes in the samples can thus change the solvus temperatures significantly. Hovis (2017) corrected his ∆*H*^mix^ values for small changes in the Al/Si distributions that occurred during homogenisation. Such a correction was, however, not applied by Bachinski and Müller (1971) to their samples. If these were characterised by Al/Si distributions that were not fully ordered, the correct solvus of the Al/Si ordered alkali feldspars would be higher.

### Diopside–jadeite solid solution

**Fig. 6:**
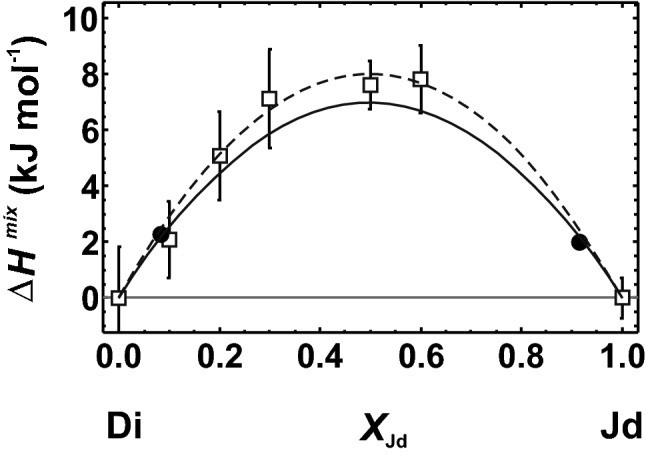
Heat of mixing (∆*H*^mix^) of the diopside (Di)–jadeite (Jd) solid solution. Experimental data from Wood et al. (1980) are indicated by open squares with error bars (1 sd). Closed circles and solid line are from LDA calculations (single defect method), where the distance between defects on M1 and M2 sites is smallest (first next nearest M1–M2 defect pair). If defect combinations with larger distances between them are considered, ∆*H*^mix^ is increased. Broken line represents ∆*H*^mix^ resulting from the mean of the five next nearest defect combinations

The diopside–jadeite binary is characterised by the coupled substitution

7 $$ {\text{Ca}}^{{{\text{M2}}}} + {\text{ Mg}}^{{{\text{M1}}}} = {\text{ Na}}^{{{\text{M2}}}} + {\text{ Al}}^{{{\text{M1}}}} , $$

where Ca is replaced by Na on the M2 site and Mg is replaced by Al on the M1 site, complicating the application of the single defect method. Strictly speaking, the single defect becomes a double defect. A previous study found that ∆*H*^mix^ of coupled substitutions depends on the defect combinations, i.e. on the distance between the two coupled defects. Such dependence was demonstrated for the Mg–Al biotites (Dachs and Benisek 2019) and is similarly expected for the pyroxene structure. The diopside structure has several possibilities for arranging the Na defect around the Al defect. There are three defect combinations with a distance of ~ 3.3 Å between them, two possibilities with a distance of 4.7 Å and many defect combinations with a distance of ~ 6 Å. If the distance between the defects on the M1 and M2 sites is smallest (first next nearest M1–M2 defect combinations), ∆*H*^mix^ is slightly smaller than the calorimetric results from Wood et al. (1980). This difference is shown in Fig. 6, where the solid line represents the results from the first next nearest defect combinations, which can be modelled with *W*^*H*^ = 27.98 kJ/mol. If defect combinations with larger distances between them are also considered, ∆*H*^mix^ becomes increasingly larger. The dashed line in Fig. 6 represents the mean of five defect combinations (*W*^*H*^ = 32.16 kJ/mol) and agrees perfectly with the calorimetric results.

To estimate the configurational entropy arising from the different numbers of defect combinations, we calculated the jadeite activity (*a*_Jd_) and compared it with the results of phase equilibrium experiments by Gasparik (1985), who studied the compositions of diopside–jadeite pyroxene in equilibrium with albite and quartz at 1200–1350 °C. The calculation of the activity of a component requires knowledge of ∆*H*^mix^, ∆*S*_vib_^exc^, and the deviation of the configurational entropy from that of a fully disordered state. To obtain ∆*S*_vib_^exc^, we applied the estimation method of Benisek and Dachs (2012) because DFT calculations for cells including enough crystallographic sites on which the substitutions take place would be a very time-consuming task for this binary. We obtained a maximum ∆*S*_vib_^exc^ = 0.64 J/mol/K, which corresponds with a *W*^*S*^_vib_ = 2.54 J/mol/K. Using these vibrational properties, the enthalpic excess quantities of the dashed line (*W*^*H*^ = 32.16 kJ/mol, Fig. 6) and an ideal activity of *a*^id^ = *X* (mixing on one site; in the case of a coupled substitution this corresponds with molecular mixing), it turns out that positive excess configurational entropies given by *W*^*S*^_config_ = 21 J/mol/K are needed to achieve good agreement with the phase equilibrium experiments by Gasparik (1985). These positive excess configurational entropies have a maximum value at *X*_Jd_ = 0.5 of *S*^exc^ = 5.25 J/mol/K. This value almost corresponds to a configurational entropy value of an additional site. Ideal mixing on one site produces a configurational entropy of *S*^cfg^ = 5.76 J/mol/K (− R (*X*_A _× ln(*X*_A_) + *X*_B _× ln(*X*_B_))) at maximum (at *X*_A_ = *X*_B_ = 0.5). This result means that the structural situation with five defect combinations corresponds with two almost independent crystallographic sites.

As already mentioned, the DFT calculations with five defect combinations agree well with the enthalpic behaviour of the samples from Wood et al. (1980). Most of these samples were synthesised at a temperature of 1350 °C. At lower temperatures, it can be expected that only the defect combinations with the smallest distances may be present in such crystals (enthalpic behaviour of the solid line in Fig. 6). The above comparison was therefore repeated using *W*^*H*^ = 27.98 kJ/mol, yielding an excess configurational entropy of *W*^*S*^_config_ = 18 J/mol/K. Now, the maximum value of the excess configurational entropy is *S*^exc^ = 4.5 J/mol/K, considerably smaller than the configurational entropy of an additional site. This demonstrates that short-range ordering exists in the diopside–jadeite solid solution at temperatures between 900 and 1350 °C. At still lower temperatures, long-range ordering occurs in crystals with compositions around Di_50_Jd_50_, generating the C2/c → P2/n phase transition of the omphacites (e.g. Fleet et al. 1978). The results of the static lattice energy calculations of Vinograd et al. (2007) agree well with our calculations, showing that short-range ordering is relevant between ca. 900 and 1300 °C (their Fig. 5).

### Diopside–CaTs solid solution

The diopside–Ca-Tschermak pyroxenes are another example of a binary characterised by a coupled substitution, i.e.

8 $$ {\text{Mg}}^{{{\text{M1}}}} + {\text{ Si}}^{{\text{T}}} = {\text{ Al}}^{{{\text{M1}}}} + {\text{ Al}}^{{\text{T}}} . $$

**Fig. 7:**
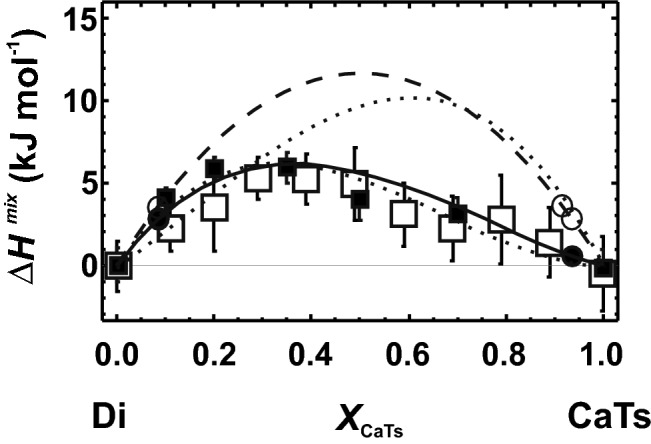
Heat of mixing (∆*H*^mix^) of the diopside (Di)–Ca-Tschermak (CaTs) solid solution. Experimental data from Benisek et al. (2007) and Newton et al. (1977), which are marked by open and closed squares with error bars, respectively (error bars represent 1 sd). Open circles and broken line represent the LDA results using the single defect method and an ordered CaTs endmember (without Al–O–Al bonds). Solid line and closed circles represent the LDA results using a partly disordered CaTs endmember (including 4 Al–O–Al linkages out of a total of 32 tetrahedral cation–anion–cation linkages, T–O–T). The CaTs-rich supercell was then constructed by inserting a substitutional defect into this partly disordered CaTs endmember, thereby reducing one Al–O–Al linkage. The dotted lines represent LDA results using other disordered CaTs endmembers. The upper dotted line shows the results, where CaTs had 8 Al–O–Al linkages out of 32 T–O–T (fully disordered) and the lower dotted line those using CaTs with 2 Al–O–Al out of 32 T–O–T. In both experiments, the CaTs-rich supercells are reduced by 1 Al–O–Al linkage through the insertion of the substitutional defect

Here, M1 and the tetrahedral site T are involved in the substitution. The tetrahedral site of the endmember CaTs (CaAlAlSiO_6_) is occupied by equal numbers of Al and Si. The two atoms are distributed somehow over one crystallographic site, implicating that CaTs is a disordered endmember. We first constructed a CaTs unit cell with alternating Al and Si on the tetrahedral chain (T–O–T); i.e. no Al–O–Al linkages were present within the tetrahedral chain. On the basis of this endmember, the resulting ∆*H*^mix^ values were large compared to measured data (Fig. 7). In a second approach, unit cells for CaTs were constructed that contained Al–O–Al linkages within the tetrahedral chain. One unit cell contained, for example, 4 Al–O–Al out of 32 T–O–T linkages to simulate a partly disordered CaTs structure. On the basis of this endmember, a supercell with a single defect was constructed, thereby removing one Al–O–Al linkage due to the insertion of Si instead of Al. Such an approach yielded perfect agreement with the measured ∆*H*^mix^ behaviour (Fig. 7). Other disordered CaTs structures were also investigated, and they showed that a partly disordered structure yielded the best agreement with the calorimetric observations.

The CaTs samples in the calorimetric studies were synthesised at temperatures between 1523 and 1673 K. Similar samples were investigated by ^29^Si MAS NMR spectroscopy (Cohen 1985; Bosenick et al. 1999; Flemming and Luth 2002), which revealed that such CaTs samples are characterised by a partly disordered Al/Si distribution on the tetrahedral site. In detail, they found that there were approximately 0.18 Al–O–Al linkages per formula unit (pfu). A fully disordered CaTs endmember would have 0.5 tetrahedral Al–O–Al, Al–O–Si, Si–O–Al, and Si–O–Si linkages pfu.

These observations are in very good agreement with our results; i.e. the CaTs structure with 4 Al–O–Al out of 32 T–O–T linkages yields the best agreement with the experimental observations. Such disordered cells contain a ratio of 0.125 Al–O–Al linkages, which corresponds to 0.25 Al–O–Al linkages pfu (because there are two T–O–T linkages in one formula unit).

The calculations showed, however, that it is not possible to ascertain the ∆*H*^mix^ behaviour unequivocally from such DFT calculations if no additional investigations are carried out. The diopside structure has six different defect combinations with the first next nearest distance between M1 and T (~ 3.3 Å). Because of this relatively large number of combinations, no DFT computations on structures with defect pairs having larger distances were performed.

### Tremolite–glaucophane solid solution

**Fig. 8:**
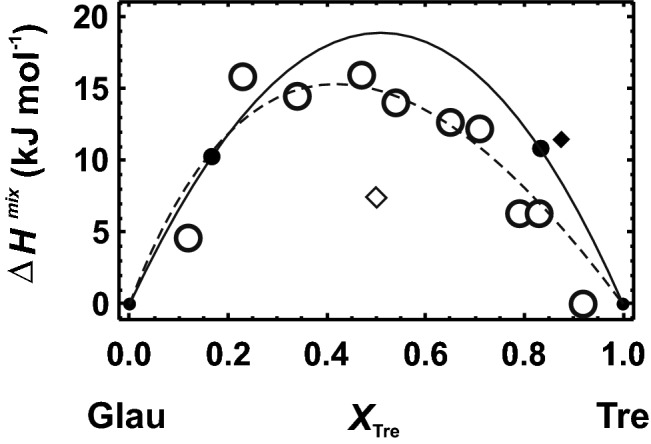
Heat of mixing (∆*H*^mix^) of the glaucophane (Glau)–tremolite (Tre) solid solution. Open circles represent the enthalpy data derived from the correlation between δ∆corr and ∆*H*^mix^ of Etzel and Benisek (2008) using the δ∆corr data of Jenkins et al. (2014) from the low wavenumber region. Solid line and solid circles represent the data from the single defect method (LDA) of this study (using the two first next nearest defect pairs). Closed diamond represents a value obtained by using one of the four second next nearest defect combinations. Open diamond is the DFT calculated ∆*H*^mix^ of a fully ordered solid solution. Broken line is from Jenkins et al. (2014)

Amphiboles have four different M sites. In tremolite, M4 is occupied by Ca and the other M positions by Mg. In glaucophane, M4 is occupied by Na, Al is fully ordered on the adjacent M2 site for temperatures lower than 1000 K (e.g. Papike and Clark 1968; Palin et al. 2003), and the other M sites are occupied by Mg. Such a highly Al-ordered state is, however, only present in alkali amphiboles because of its monovalent M4 cation. On the other hand, in calcic amphiboles, Al is highly disordered over the M2 and M3 sites (Palin et al. 2003). For the sake of simplicity, the glaucophane defect in the tremolite-rich phase was constructed by positioning Na on M4 and Al only on M2. The tremolite defect in the glaucophane-rich phase consisted of Ca on M4 and Mg on M2. There are two defect combinations with the first next nearest M4–M2 distance and four combinations with the second next nearest distance. The latter yielded slightly larger ∆*H*^mix^ values than the former. The first next nearest combinations were described by a Margules mixing model using *W*^*H*^_TreGlau_ = 72.91 kJ/mol and *W*^*H*^_GlauTre_ = 78.58 kJ/mol. This ∆*H*^mix^ behaviour is plotted in Fig. 8, where it is compared with enthalpic data obtained from the relation between line broadening in IR spectra (δ∆corr) and ∆*H*^mix^ according to Etzel and Benisek (2008) using the δ∆corr data of Jenkins et al. (2014). Figure 8 also shows the ∆*H*^mix^ behaviour from Jenkins et al. (2014) derived from phase equilibrium experiments performed on the miscibility gap. Jenkins et al. (2014) also investigated δ∆corr of their synthetic samples and used these values to extract ∆*H*^mix^ after Etzel and Benisek (2008). They obtained maximum values of 14 kJ/mol, if δ∆corr is taken from the low wavenumber region, corresponding to a symmetrical *W*_TreGlau_ of 56 kJ/mol (based on ∆*H*^mix^ = *X*_Tre_*X*_Glau_*W*_TreGlau_ and *X*_Tre_ = *X*_Glau_ = 0.5). Their experimentally derived *W*_TreGlau_ = 70 kJ/mol leads to a solvus with an apex of around 800 °C (Jenkins et al. 2014, their Fig. 4). The statement of Jenkins et al. (2014, p. 739) that a maximum ∆*H*^mix^ = 14 kJ/mol would lead to an unrealistically high critical temperature of the solvus (~ 4000 °C) is obviously wrong.

**Fig. 9:**
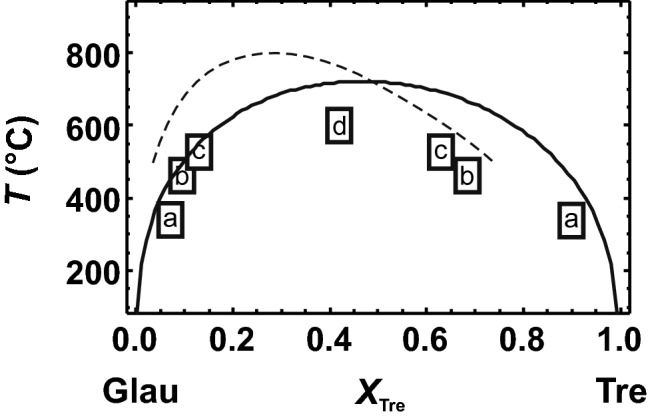
Miscibility gap of the glaucophane (Glau)–tremolite (Tre) join at ~ 1.8 GPa. Solid line represents the results derived from the calculated enthalpy, entropy and volume data of this study. Open rectangles represent data based on coexisting natural amphiboles from a Black (1973), b Schliestedt (1980), c Reynard and Ballevre (1988) and d Maresch et al. (1982). Broken line was taken from Jenkins et al. (2014)

The DFT calculations yielded slightly larger ∆*H*^mix^ values than the data derived from δ∆corr and from the phase equilibrium experiments (Fig. 8). To calculate the solvus, ∆*S*_vib_^exc^ was again calculated according to Benisek and Dachs (2012), yielding *W*^*S*^_vib_ = 11.73 J/mol/K. The configurational entropy was calculated assuming mixing on four sites. The resulting solvus is shown in Fig. 9, where it is compared to the solvus from Jenkins et al. (2014) and coexisting natural amphiboles. The solvus based on the DFT calculations shows a similar height, but an asymmetry that does not agree with the natural and experimental evidence. This result is most likely a consequence of the assumed simple model for the configurational entropy. In the tremolite-rich region, Al may also occupy the M3 site, increasing the configurational entropy, whereas the glaucophane-rich region may be characterised by a stricter ordering not correctly described by mixing on four sites.

## Conclusions

The DFT-calculated mixing properties of all investigated solid solutions are summarised and, if possible, compared with measured ones in Table 1. This study showed that DFT calculations in combination with the single defect method can generate accurate heat of mixing values, amazingly accurate for simple solid solutions. This method allows the investigation of this property considerably faster than calorimetric methods (the DFT calculations are performed within weeks compared to years for calorimetric work). In cases where ordering effects play a dominant role (mixing on two different crystallographic sites), additional information is needed to predict valuable results. If this method can be expanded to binary systems containing Fe remains the subject of further studies and is tested presently.

**Table 1:** Excess thermodynamic properties of the investigated binaries

	Observed	DFT-calculated					*a*^id^
	max∆*H*^mix^ (kJ/mol)	max∆*H*^mix^ (kJ/mol)	*W*^*H*^_AB_ (kJ/mol)	*W*^*H*^_BA_ (kJ/mol)	*W*^*S*^^vib^_AB_ (J/mol/K)	*W*^*S*^^vib^_BA_ (J/mol/K)	
NaCl–KCl	4.5^b^	4.75^a^	16.45^a^	22.01^a^	8.73^c^	8.73^c^	*a*_NaCl_^id^ = *X*_Na_ *a*_KCl_^id^ = *X*_K_
Pyrope–grossular (Py–Gr)	9.6^d^	9.3^a^	32.72^a^	44.50^a^	− 7.4^e^	27.9^e^	*a*_Py_^id^ = *X*_Mg_^3^ *a*_Gr_^id^ = *X*_Ca_^3^
MgO–CaO	–	19.2^a^	65.24^a^	86.84^a^	10.8^a^	10.8^a^	*a*_MgO_^id^ = *X*_Mg_ *a*_CaO_^id^ = *X*_Ca_
Low albite–microcline (Ab–Mic)	8.1^f^	6.5^a^	20.59^a^	31.30^a^	9.1^g^	9.1^g^	*a*_Ab_^id^ = *X*_Na_ *a*_Mic_^id^ = *X*_*K*_
Diopside–jadeite (Di–Jd)	7.8^h^	7.0^a^	27.98^a^	27.98^a^	2.54^i^	2.54^i^	*a*_Di_^id^ = *X*_Ca_^M2^*X*_Mg_^M1^ *a*_Jd_^id^ = *X*_Na_^M2^* X*_Al_^M1^ *T* > 1350 K^n^
Diopside–CaTs (Di–CaTs)	6.0^k^	6.0^j^	7.70^j^	37.44^j^	0^l^	0^l^	*a*_Di_^id^ = *X*_Mg_^M1^ (*X*_Si_^T^)^2^ *a*_CaTs_^id^ = 4*X*_Al_^M1^*X*_Al_^T^*X*_Si_^T^ + SRO^o^
Glaucophane–tremolite (Glau–Tre)	16.0^m^	18.5^a^	78.58^a^	72.91^a^	11.73^i^	11.73^i^	*a*_Glau_^id^ = (*X*_Na_^M4^)^2^(*X*_Al_^M2^)^2^ *a*_Tre_^id^ = (*X*_Ca_^M4^)^2^(*X*_Mg_^M2^)^2^ + SRO^p^

max∆*H*^mix^ is the heat of mixing at the mole fraction where it is at its maximum. DFT-derived values are compared to observed ones. *W*^*H*^_AB_ and *W*^*H*^_BA_ are the enthalpic interaction parameters of the asymmetric Margules mixing model, ∆*H*^mix^ = (1 − *X*_B_) *X*_B_^2^*W*^*H*^_AB_ + (1 − *X*_B_)^2^*X*_B_*W*^*H*^_BA_, of the A–B binary. *W*^*S*^^vib^_AB_ and *W*^*S*^^vib^_BA_ are the interaction parameters for the vibrational excess entropy, ∆*S*_vib_^exc^ = (1-*X*_B_) *X*_B_^2^*W*^*Svib*^_AB_ + (1-*X*_B_)^2^*X*_B_*W*^*Svib*^_BA_. To calculate the excess Gibbs energy of mixing, use *W*^*G*^ = *W*^*H *^– *T W*^*S*^ and *G*^exc^ = (1 − *X*_B_) *X*_B_^2^*W*^*G*^_AB_ + (1 − *X*_B_)^2^*X*_B_*W*^*G*^_BA_. Ideal mixing is defined in the last column (*a*^id^). To define the solvus, the use of a configurational excess entropy is needed in most cases, which is, however, not listed because it is only valid at the solvus temperatures and may vanish at higher temperatures

^a^DFT methods using LDA functional, this study

^b^Solution calorimetry (Barrett and Wallace 1954)

^c^Low-temperature calorimetry (Benisek and Dachs 2013)

^d^Solution calorimetry (Newton et al. 1977)

^e^Low-temperature calorimetry (Dachs 2006)

^f^Solution calorimetry (Hovis 2017)

^g^Low-temperature calorimetry (Benisek et al. 2014)

^h^Solution calorimetry (Wood et al. 1980)

^i^According to Benisek and Dachs (2012)

^j^DFT methods using a partly disordered CaTs endmember

^k^Solution calorimetry (Newton et al. 1977; Benisek et al. 2007)

^l^Low-temperature calorimetry (Etzel et al. 2007)

^m^Derived from line broadening in IR according to Etzel and Benisek (2008)

^n^At *T* < 1350 K, short-range ordering is present and at *T* < 1000 K, long-range ordering exists (Fleet et al. 1978)

^o^Short-range ordering is present; see for example Benisek et al. (2007)

^p^Possible short-range ordering is present, this study

## Electronic supplementary material

Below is the link to the electronic supplementary material.

Supplementary file1 (PDF 97 kb)

Supplementary file2 (PDF 101 kb)
