# Supplementary material for: Excess enthalpy of mixing of mineral solid solutions derived from density-functional calculations
Source: Phys Chem Miner. 2020 Feb 17;47(3):15. doi: 10.1007/s00269-020-01085-8 (PMC7024695; doi:10.1007/s00269-020-01085-8)

Appendix A:
NaCl – KCl solid solution:
Margules parameters (*W*) as a function of the distance between single defects in neighbouring cells from the study "Excess enthalpy of mixing of mineral solid solutions derived from density-functional calculations" published by Artur Benisek and Edgar Dachs in Physics and Chemistry of Minerals.

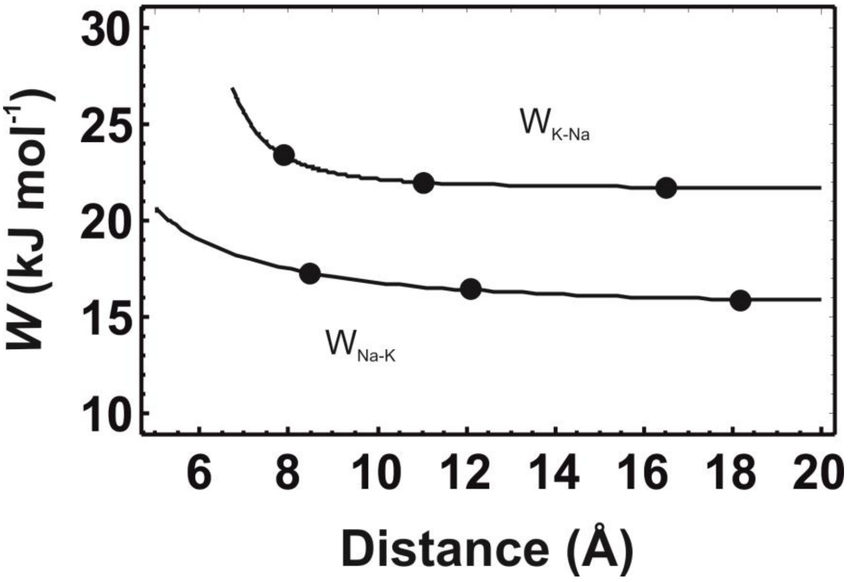

Supplement: Supplementary file 1 — Supplementary file1 (PDF 97 kb) [file 269_2020_1085_MOESM1_ESM.pdf]
