# Supplementary material for: Excess enthalpy of mixing of mineral solid solutions derived from density-functional calculations
Source: Phys Chem Miner. 2020 Feb 17;47(3):15. doi: 10.1007/s00269-020-01085-8 (PMC7024695; doi:10.1007/s00269-020-01085-8)

Appendix B:
Solvus of the NaCl – KCl solid solution from the study "Excess enthalpy of mixing of mineral solid solutions derived from density-functional calculations" published by Artur Benisek and Edgar Dachs in Physics and Chemistry of Minerals.
Red and blue data points are from Barrett and Wallace (1954) and Vesnin and Zakovryashin (1979), respectively. The curve is calculated using the heat of mixing data from this study and the vibrational excess entropy data from Benisek and Dachs (2013).

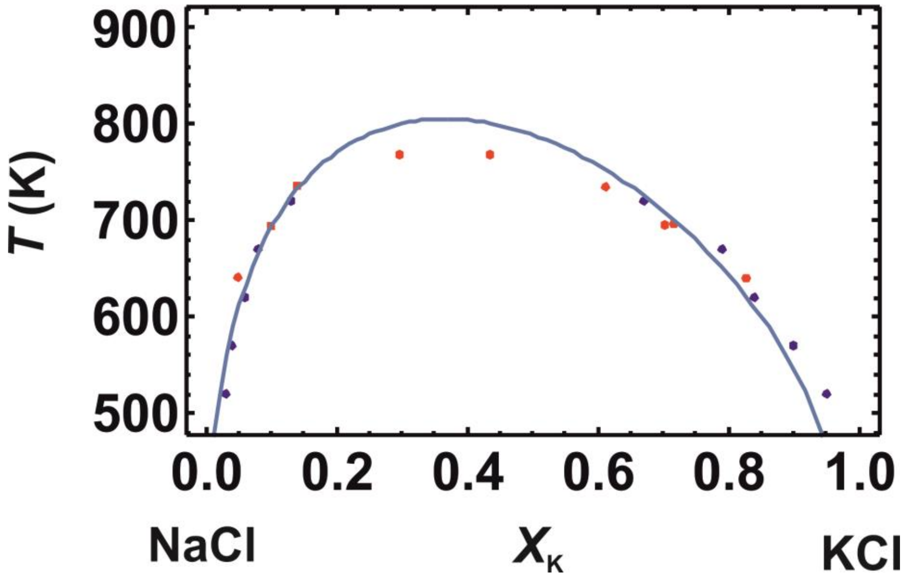

Supplement: Supplementary file 2 — Supplementary file2 (PDF 101 kb) [file 269_2020_1085_MOESM2_ESM.pdf]
